# The impact of early life stress and schizophrenia on motor and cognitive functioning: an experimental study

**DOI:** 10.3389/fnint.2023.1251387

**Published:** 2023-10-19

**Authors:** Fredrick Otieno Oginga, Thabisile Mpofana

**Affiliations:** ^1^Department of Physiology, School of Laboratory Medicine and Medical Sciences, University of Kwa-Zulu Natal, Durban, South Africa; ^2^Department of Human Physiology, School of Bio-molecular & Chemical Sciences Mandela University, University Way, Summerstrand, Gqeberha, South Africa

**Keywords:** early life stress, schizophrenia, psychopathology, anxiety, parental mental illness, cognitive (spatial memory task, learning & spatial navigation)

## Abstract

**Background:**

Early life stress (ELS) and parental psychopathology, such as schizophrenia (SZ), have been associated with altered neurobiological and behavioral outcomes later in life. Previous studies have investigated the effects of ELS and parental SZ on various aspects of behavior, however, we have studied the combined effects of these stressors and how they interact, as individuals in real-life situations may experience multiple stressors simultaneously.

**Objective:**

The aim of this study was to investigate the impact of ELS and schizophrenia on locomotor activity, anxiety-like behavior, exploratory tendencies, and spatial memory in Sprague Dawley (SD) rats.

**Methods:**

Male and female SD pups were randomly assigned to eight groups: control, ELS, schizophrenia, and ELS + schizophrenia. ELS was induced by prenatal stress (maternal stress) and maternal separation (MS) during the first 2 weeks of life, while SZ was induced by subcutaneous administration of ketamine. Behavioral tests included an open field test (OFT) for motor abilities and a Morris water maze (MWM) for cognitive abilities. ANOVA and *post hoc* Tukey tests were utilized to analyze the data.

**Results:**

Our results show that ELS and parental psychopathology had enduring effects on SZ symptoms, particularly psychomotor retardation (*p* < 0.05). The OFT revealed increased anxiety-like behavior in the ELS group (*p* = 0.023) and the parental psychopathology group (*p* = 0.017) compared to controls. The combined ELS and parental psychopathology group exhibited the highest anxiety-like behavior (*p* = 0.006). The MWM analysis indicated impaired spatial memory in the ELS group (*p* = 0.012) and the combined ELS and parental psychopathology group (*p* = 0.003) compared to controls. Significantly, the exposure to ELS resulted in a decrease in the population of glial fibrillary acidic protein-positive (GFAP^+^) astrocytes. However, this effect was reversed by positive parental mental health.

**Conclusion:**

Our findings highlight the interactive effects of ELS and parental psychopathology on anxiety-like behavior and spatial memory in rats. ELS was linked to increased anxiety-like behavior, while SZ was associated with anhedonia-like behavior. Positive parenting augments neuroplasticity, synaptic function, and overall cognitive capacities.

## 1. Introduction

Stress is the body’s response to various internal or external stimuli, often stressors that disrupt the organism’s physiological equilibrium or homeostasis (Chrousos and Gold, 1992; McEwen and Akil, 2020). When faced with a stressor, the body activates its stress response system, which triggers a cascade of physiological and hormonal changes (Chrousos, 2009). This response is primarily regulated by the hypothalamic-pituitary-adrenal (HPA) axis and the sympathetic-adrenal-medullary (SAM) system (Stults-Kolehmainen et al., 2014; Kuhlman et al., 2018).

Early life stress (ELS) encompasses detrimental occurrences or difficult situations that occur during the initial stages of development, commonly in childhood or infancy, that have the potential to disrupt the physical, emotional, or social well-being of the child, for example, maltreatment, neglect, separation, parental loss, extreme poverty, starvation, domestic/community/school violence (Saleptsi et al., 2004). Importantly, the impact of ELS extends beyond the immediate context. Recent studies have revealed that intricate cognitive and emotional functions linked to brain regions with extended postnatal development are particularly at risk due to ELS. Lastly, enduring deficits, particularly in emotional realms, persist long after ELS and could heighten the chances of future psychopathology. Considering this, the utilization of maternal separation (MS), gestational stress (GS), and chronic unpredictable stress (CUS) as models for early-life stress in rodent research has gained considerable traction. These models reveal that progeny subjected to varying stressors within this paradigm tend to exhibit submissive and passive stress responses during their adult phases (Cabrera et al., 1999; Gardner et al., 2005; Wilson et al., 2013; Abramova et al., 2021). Early life stress has consistent effects on neural development. It disrupts the functioning of crucial systems like the HPA, autonomic nervous system (ANS), and immune system (Alkon et al., 2014; Danese and Lewis, 2017; Koss and Gunnar, 2018), which are essential for shaping psychological and behavioral responses to environmental challenges (McEwen, 2019). These effects are rooted in changes within neural circuits associated with stress response, including the prefrontal cortex, hippocampus, amygdala, and striatal circuits (Fareri and Tottenham, 2016; McEwen and McEwen, 2017).

Schizophrenia (SZ) is a prominent neuropsychiatric disorder, marked by cognitive, emotional, and perceptual disruptions that profoundly affect individuals’ thoughts, behaviors, and overall functioning. Its intricate origins involve genetic, neurobiological, environmental, and psychosocial factors (Faden, 2019; Pan, 2022). Numerous candidate genes related to neurotransmitter regulation, synaptic plasticity, and neurodevelopment contribute to its complexity. Notably, disturbances in dopaminergic signaling, particularly within the mesolimbic pathway, correlate with positive symptoms like hallucinations and delusions. The interplay of these factors underscores the multifaceted nature of the disorder (Plomin and Daniels, 1987; Zamanpoor, 2020; Konopaske and Coyle, 2022). Conversely, parental psychopathology such as SZ refers to cases where children are born to parents that experience this mental disorder. In these situations, the offspring may have an increased risk of inheriting a genetic predisposition for SZ. Additionally, environmental factors and the quality of parental care may also play a role in influencing the child’s development and susceptibility to mental disorder later in life (Bakoula et al., 2009; Breaux et al., 2014; Doyle, 2016). Research on ELS in rodents has provided valuable insights into the underlying mechanisms of stress-related disorders in humans (Roth, 2012; Phillips and Roth, 2019; Alfonso and Schulze, 2021).

ELS represents a critical phase in an individual’s developmental journey, with the potential to significantly impact motor and cognitive functioning. This developmental window is characterized by heightened neuroplasticity and vulnerability, rendering it a pivotal period for shaping cognitive trajectories (Pechtel and Pizzagalli, 2011). Astrocytes, which are integral components within the tripartite synapse structure, assume pivotal roles in the regulation of synaptic transmission dynamics. Empirical evidence suggests astrocyte involvement in psychiatric disorders (Cotter et al., 2001; Bender et al., 2016), exemplified by altered morphology in mood disorders and their role in anxiety-like behavior through amygdala activation. The hippocampus, central to anxiety, features granules and CA1 pyramidal cells linked to anxiolytic effects (Jimenez et al., 2018). While neural circuits implicate the hippocampus in anxiety behaviors, the contribution of hippocampal astrocytes remains unexplored.

The objective of this study was to utilize a rat model to investigate the impact of early life stress and parental psychopathology on motor and cognitive functioning. The rationale for combining early life stress and parental psychopathology in this rat model study is to simulate the multifaceted nature of human experiences and examine potential interactive effects between these stressors on memory, motor, and learning. This investigation aimed to explore the potential involvement of neurodegeneration in the mechanisms underlying the impact of ELS and SZ on motor and cognitive abilities.

## 2. Materials and methods

### 2.1. Experimental animals and treatment

The Sprague Dawley (SD) strain is widely recognized for its susceptibility to a range of health ailments, such as stress, cancer, diabetes, and cardiovascular disorders, which closely mirror human conditions (Brower et al., 2015). In alignment with this motivation, a total of 24 SD rats, comprising 16 nulliparous females in the fertile phase and eight stud males, were procured from the Biomedical Resource Unit (BRU) breeding center at the University of KwaZulu-Nata (UKZN). The animals were bred and raised within the controlled environment of the Animal House, located at the UKZN’s BRU. For this study, a pure breed of the SD strain was used to ensure consistency and uniformity in the experimental cohort. All animals were subjected to standard laboratory conditions, including being housed in pairs within standard cages (29 cm by 22 cm by 14 cm). The temperature was maintained at a constant level, and a 12 h dark photoperiod was implemented. The rats were provided with unrestricted access to both food and water *ad libitum*. The present study adhered to ethical guidelines and obtained prior approval before commencing the research. The study protocol was assigned the reference number AREC/00003119/2021.

### 2.2. Animal housing and surgery

The study involved mating a total of 16 nulliparous females in the fertile phase and eight proven stud male rats to obtain 64 pups for the experiments. After the animals were collected from the BRU, the females were paired in cages for 1 week to acclimatize and minimize stress and to synchronize their estrous cycles. The stud males were individually housed for 1 week prior to the mating to build up sperm count and maximize their fertility. Vaginal smears were taken to assess their estrous cycle, and when they were at proestrus, two nulliparous females were transferred into a stud male cage. On the subsequent morning, the researchers examined the presence or absence of an ejaculatory plug within the vaginal cavity, as outlined in the study conducted by Paccola et al. (2013). Upon the discovery of a plug, the female was promptly confined within a separate cage, and subsequently labeled with a designated breeding date. In this study, the initiation of the plug formation was designated as the starting point of gestation, denoted as day one. After mating, males were removed from the cages and euthanized to avoid stalk contamination.

### 2.3. Groupings and stressors

The study consisted of eight distinct groups, each comprising eight pups. On the day of delivery, four pups were randomly selected from each dam and labeled accordingly for proper follow-up. The distribution of these groups and the corresponding number of pups are presented in Table 1. Dams and any additional pups were euthanized post-weaning.

**Table 1:** Experimental design and behavioral tests in early life stress (ELS) study.

Groups	Number of pups (*n*)	Treatment conditions	Stressors
Control	8	No stressor	No stressor
Prenatal maternal stress + ketamine-injected pups (group 1)	8	Early life stress + parental psychopathology	Re-strainer maternal stress and ketamine injection on the pups PND-7, 9, 11 & 15
Prenatal maternal stress (group 2)	8	Parental psychopathology only	Re-strainer maternal stress
Maternally separated pups (group 4)	8	Early life stress only	Maternal separation for 3 h from PND-1 to PND-14
Maternal separated + ketamine-injected pups (group 5)	8	Early life stress only	Maternal separation for 3 h from PND-1 to PND-14 and ketamine injection on the pups PND-7, 9, 11 and 15
Ketamine-injected parent (group 6)	8	Parental psychopathology only	Ketamine will be injected into the parents on PND-2, 4 to 6
Ketamine parent + ketamine pups (group 7)	8	Early life stress + parental psychopathology	Ketamine will be injected into the parents on PND-2, 4 to 6 & ketamine pups
Ketamine (positive control) (group 8)	8	Early life stress only	Ketamine injection on the pups PND-7, 9, 11 and 15

The table outlines different treatment groups and the specific stressors the animals were exposed to.

#### 2.3.1. Gestational stress (maternal restrainer stress)

To mimic human prenatal stress in this study, the pregnant rats were subjected to daily GS induction from days 15 to 18. The experimental procedure consisted of subjecting the pregnant dams to a stress protocol, which entailed confining them within transparent cylindrical restrainers (8–10 cm in diameter and 20–24 cm in length) for 45 min (Murmu et al., 2006). This confinement took place in a well-illuminated environment maintained at a temperature range of 21°C–22°C. The dimensions of the restrainers were modified in accordance with the body mass of the laboratory rats throughout the gestational phase of the experimental investigation, which was anticipated to span a duration of approximately 21 to 23 days.

#### 2.3.2. Maternal and child psychopathology

This study examined the association between parental psychopathology and the likelihood of passing down mental health issues. Offspring susceptibility to disorders arises from a mix of genetic, environmental, and psychosocial factors (McKinney and Stearns, 2021; Sorcher et al., 2022). We used an experimental model, inducing SZ in dams from post-natal day 1 to 7. The dams and their offspring were grouped per Table 1 conditions. We utilized ketamine, an N-methyl-D-aspartate (NMDA) receptor antagonist, which mimics cognitive, positive, and negative symptoms of SZ (Neill et al., 2010; Kocsis et al., 2013). Dams received (30 mg/kg, i.p) daily doses for five consecutive days while the control group received saline (0.5 mL/kg, i.p) (Chan et al., 2008), and pups were injected with ketamine (16 mg/kg, subcutaneously) three times a week (on Mondays, Wednesdays, and Fridays). This treatment protocol was initiated on postnatal day 1 and continued until postnatal day 14. The selection of dosage, route of administration, and injection schedule were determined through a comprehensive analysis of prior scientific investigations that have reported the induction of psychotic-like alterations subsequent to a 5 days regimen of ketamine treatment (Schobel et al., 2013; Koh et al., 2016; Lisek et al., 2017). On postnatal day 21, the pups were weaned while the maternal subjects were subjected to euthanasia. The animals were left undisturbed until postnatal day 60, at which point they were subjected to behavioral testing.

#### 2.3.3. Maternal separation

MS is a research paradigm that is frequently employed in animal studies to examine the impact of ELS and the lack of MC on the development of offspring. According to a recent study conducted by Mejía-Chávez et al. (2021), it has been observed that MS has the potential to disrupt the attachment bond between the mother and offspring. To study MS effects, we separated dams from their offspring for 3 h daily (9 a.m. to 12 p.m.) from postnatal days 1 to 14. The dams were relocated from their original cages. The pups were subsequently removed from the experimental rooms and relocated to a distinct room (to establish a barrier to communication between the offspring and their respective mothers).

**Figure 1:**
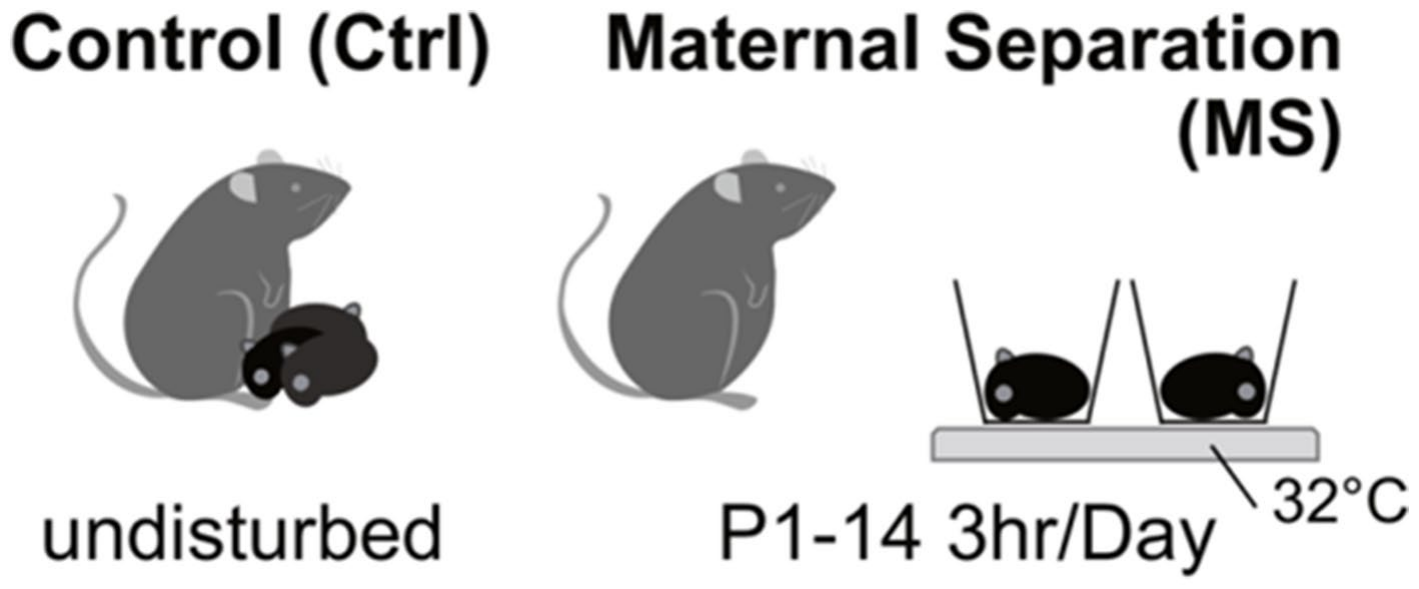
Illustration of the maternal separation paradigm in early life stress study.

**Figure 2:**
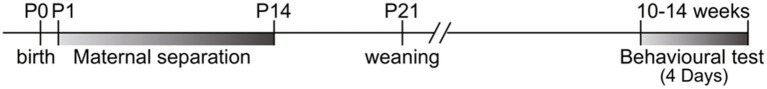
Timeline of maternal separation, weaning, and behavioral test schedule in ELS study.

Following a period of 3 h (9 a.m. to 12 p.m.), the pups were reintroduced to the designated animal room, and the dams returned to their respective home cages (Vetulani, 2013; Kestering-Ferreira et al., 2021). Figure 1 presents a visual representation showcasing the MS paradigm utilized in the investigation of ELS (Figure 2).

### 2.4. Behavioral test

#### 2.4.1. Open field test

The OFT is a valuable and widely used behavioral assay that is fundamental for assessing various aspects of rodent behavior, offering insights into multiple psychological and physiological dimensions (Seibenhener and Wooten, 2015; Kraeuter et al., 2019). In this test, the animal’s spontaneous locomotor activity and exploratory behavior are measured in a novel open arena. The experiment used a 100 cm × 100 cm rectangular enclosure with 40 cm high plywood walls. This box had a clean, opaque plastic floor, which was divided into 25 even squares by a white grid. A 60 W bulb, strategically located 100 cm above the enclosure’s center, served as the main light source. Before introducing each animal, the apparatus was cleaned using a 10% cider vinegar solution. The rat was strategically positioned within one of the corners of the designated apparatus. Subsequently, their behavioral patterns and activities were observed and recorded for 5 min. The following parameters were measured, i.e., the total distance traveled (cm), the number of entries to the center of the field, serving as an indicator of the rat’s exploratory behavior, and willingness to venture into unfamiliar territory. Furthermore, we measured the time spent in the center of the field (seconds), allowing for anxiety evaluation. The experimental methodology described herein has been previously employed in various scientific investigations (Gould et al., 2009; Võikar and Stanford, 2023).

#### 2.4.2. Morris water maze

**Figure 3:**
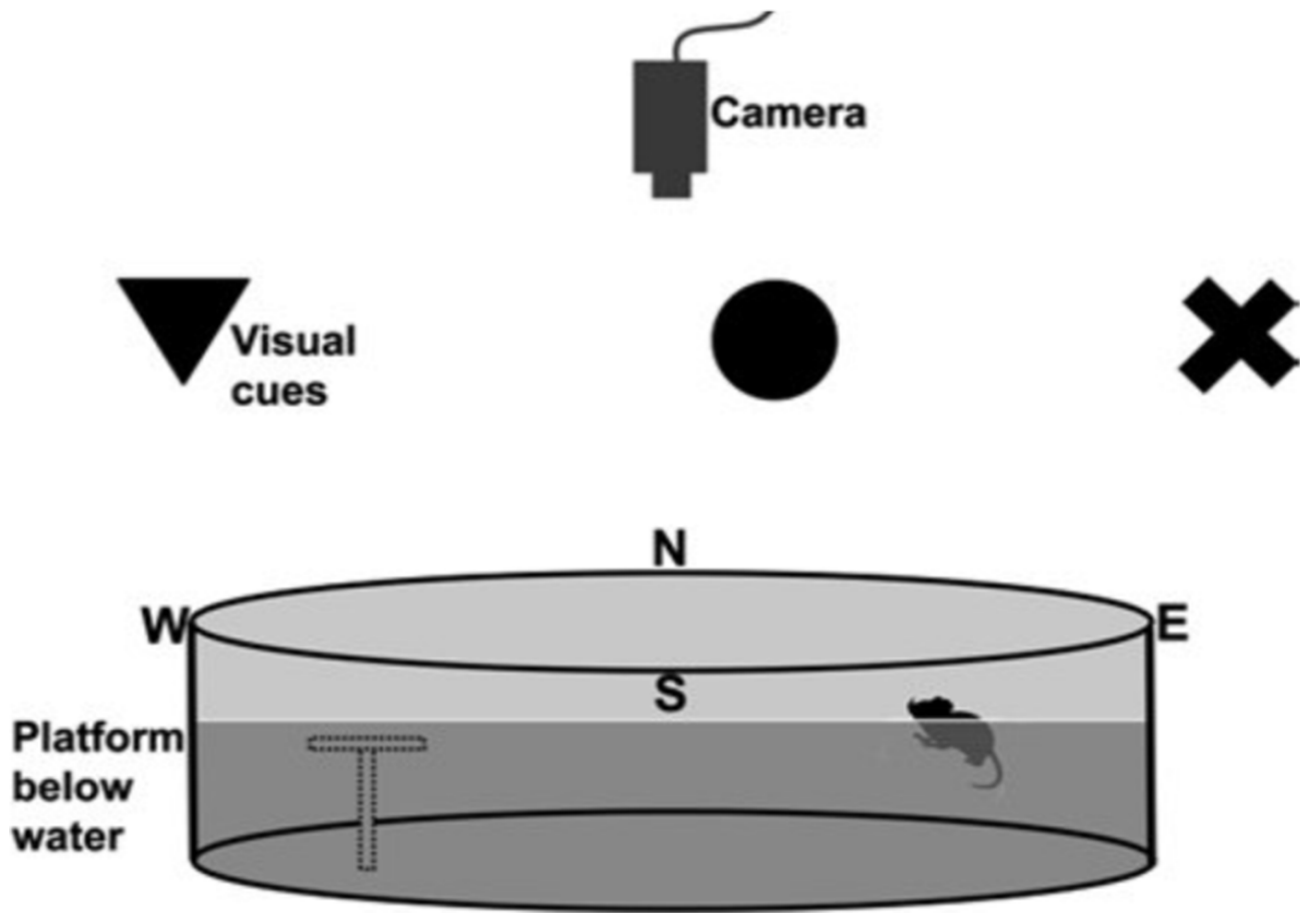
Illustration of the setup of the Morris water maze, which consisted of a circular pool placed in a room with external cues for learning. An overhead camera recorded the rats’ swimming paths. To accommodate animals with reduced visual acuity, high-contrast large shapes served as cues against the background. The transparent platform, depicted as a gray outline, remained invisible to the rats. Starting from the north (N), south (S), east (E), and west (W) points, the rats used cues to navigate and find the hidden platform (Sherrard, 2011; Barnhart et al., 2015).

Spatial learning and memory cognitive abilities of the rats were evaluated at postnatal day 60 through the utilization of the Morris water maze (MWM) paradigm (Vorhees and Williams, 2006; Nunez, 2008; Barnhart et al., 2015). The MWM entailed subjecting the laboratory rats to a pool filled with water that had a milky appearance. The primary objective was for the rats to navigate and locate a submerged escape platform. The experimental pool utilized in this study had a circular shape with a diameter of 6 feet and a depth of 3 feet. The water temperature within the pool was maintained at a constant value of 26°C throughout the experiment. The experiment had a training phase and a test phase. During the training phase, the platform was deliberately elevated by a marginal distance of one inch above the water surface. This was done to effectively direct the rats toward the platform, serving as the designated exit point (see Figure 3).

Following the completion of the training phase, the experimental platform was positioned approximately one inch below the surface of the opaque water. The objective of this experiment was to assess the rats’ ability to retain and recall the spatial information associated with the platform’s location. Each rat was subjected to three consecutive trials; the initial starting positions were randomly assigned as either north, south, east, or west. The rats were placed in water and allowed to swim for up to 60 s. If the rat failed to locate the platform within 60 s, an appropriate intervention was promptly administered, ensuring the rat’s safety and the time taken was recorded as 1 min. After three trials, the rat was dried utilizing a towel (Nunez, 2008; Salman et al., 2019). These parameters measured included the latency to reach the platform, the time spent in the target quadrant, and the swim speed.

### 2.5. Sacrifice and neurochemical analysis

#### 2.5.1. Animal sacrifice and tissue collection

The animals included in this study were subjected to humane care in accordance with the guidelines established by the Institutional Animal Use and Care Committee of the School of Laboratory Medicine and Medical Science at UKZN. The study protocol was assigned the reference number AREC/00003119/2021. Prior to sacrifice, animals were anesthetized using a combination of ketamine and xylazine or Halothane, inducing a state of deep unconsciousness to minimize any potential distress or pain. Once the desired level of anesthesia was confirmed, transcardial perfusion or the decapitation procedure was performed. The sacrifice was done by trained personnel experienced in the procedures, maintaining a focus on precision and compassion. Detailed records of each sacrificing event, including animal identification, date, time, and personnel involved, were documented for regulatory compliance and transparency. The date of sharpening of the guillotine was not more 3 than weeks from the decapitation date.

#### 2.5.2. Transcardial perfusion fixation

Twenty-four hours following the conclusion of the behavioral assessment, a cohort of SD rats was injected with ketamine (80 mg/kg, i.p) and xylazine (10 mg/kg, i.p) using a 27-gauge needle and a 1 cc syringe. After the injection, the rats were further anesthetized using sodium pentobarbital according to their body weights.

The rats were perfused using a solution of phosphate-buffered saline (PBS) and 4% paraformaldehyde (PFA). To optimize perfusion, a minor laceration was created at the caudal aspect of the left ventricle following the exposure of the heart. A perfusion needle (15 gauge) with either a blunt or olive tip, specifically designed for this purpose, was carefully inserted through a surgical incision. The needle was inserted into the ascending aorta. If required, supplementary anesthesia was administered to maintain the rats at an optimal level of surgical anesthesia.

#### 2.5.3. Decapitation, post fixation, tissue collection, and storage

At postnatal day 60, the SD rats were anesthetized using Halothane in accordance with their body weight. The head of each rat was separated from the body using a sharp guillotine. Blood samples were collected from the jugular vein and stored in vacutainers for further analysis. To expose the skull, a midline incision was made along the skin from the neck to the nose, and the neck muscles were trimmed to reveal the base of the skull. An identical incision was made on the opposite side, extending to the posterior edge of the skull surface.

Subsequently, the brain was carefully removed from the skull. The brain was then placed in a vial containing a fixative solution, ensuring that the volume of the fixative was at least 10 times greater than the brain’s size. The vial was intermittently swirled while kept in the fixative solution for 24 h at a temperature of 4°C. The brain was rinsed with phosphate-buffered saline through three media exchanges, with swirling performed after each exchange. The harvested hippocampal astrocytes were stored in phosphate-buffered saline with sodium azide and kept at a temperature of 4°C for further use (van Rijn et al., 2011).

#### 2.5.4. Astrocyte cell cultures

##### 2.5.4.1. Tissue preparation

Following euthanasia, the rats were subjected to transcardial perfusion using a solution of 0.1 M phosphate buffer (PB) with a volume of 50 mL. This was subsequently followed by perfusion with a fixative solution composed of 4% paraformaldehyde in 0.1 M PBS (pH 7.4), with a total volume of 100 mL. The fixative solution was maintained at a low temperature, specifically ice-cold, to ensure optimal preservation of the samples. The cerebral organs were meticulously removed from the cranium, followed by their exposure to a subsequent fixation procedure within the identical solution used for initial fixation, spanning a time period of 3 h.

To acquire coronal sections of the brain that encompassed the hippocampus, we employed a vibratome (model VT1200, manufactured by Leica). The following sections were preserved in a 0.1 M PB solution supplemented with 0.1% sodium azide for the purpose of immunofluorescence labeling. The sections had a thickness of 60 micrometers. Alternatively, some sections were stored in a 0.1 M PB solution without any additives for the purpose of intracellular fills, with a section thickness of 100 micrometers.

##### 2.5.4.2. Cresyl violet staining and volume estimation

In this study, cresyl violet was used as a staining technique to effectively visualize and demarcate the boundaries of the hippocampus within the examined sections. An anatomical microscope with a magnification of 1.25× was used to determine the number of grid points within the hippocampus. The estimation of the total volume of the hippocampus was conducted using Cavalieri’s principle, as described by Gundersen et al. (1988) and von Bartheld (2002). In this principle, the volume (*V*) of either the hippocampus or its sub-regions was determined. The thickness (*t*) of the tissue block used for analysis was set at 0.6 mm. Each grid point was associated with an area [*a*(*p*)] of 0.09 mm^2^. The total number of grid points (Σ*P*) present in the hippocampus per rat was calculated.

##### 2.5.4.3. Immuno-histochemical labeling

The tissue slices were subjected to a sequential washing protocol, which comprised three consecutive 10 min washes in a 0.1 M PB solution supplemented with 0.5% Triton X-100 (TX). The washing process was followed by a blocking step (3 h) using PB solution, which consisted of 2% normal goat serum (NGS), 3% bovine serum albumin, and 0.3% Triton X-100 (TX).

Tissues were then incubated for 48 h at 4°C. During this incubation, a mouse monoclonal antibody (MAB360, Millipore) specifically designed to target GFAP was utilized. The antibody was diluted to a ratio of 1:10,000 in a working buffer solution composed of PB containing 2% NGS (normal goat serum) and 0.3% TX (Triton X). Following the incubation period, the sections were subjected to a series of three 10 min washes in the working buffer, then transferred to a working buffer maintained at a temperature of 25°C. The working buffer contained a 1:500 dilution of goat anti-mouse IgG conjugated to Alexa Fluor 633 (Inqaba Biotechnical SA). After a 3 h incubation period, the sections were subjected to a subsequent 15 min incubation step at 25°C in a working buffer solution that was prepared by diluting DAPI (Sigma) at a ratio of 1:2000.

The sections were washed and then carefully mounted in Prolong Gold antifade reagent. Astrocytes and principal neurons were identified using a confocal microscope, which relies on the distinct characteristics of their soma in terms of size and shape.

Immunofluorescence labeling was used to detect the presence of the astroglial marker S100b. To achieve this, free-floating sections were subjected to an overnight incubation with a rabbit anti-S100b antibody (Switzerland) at a dilution of 1:400. Tissues were then incubated for 1 h with 20 mg/mL carbocyanine (Cy) 2-conjugated donkey anti-rabbit IgG (Dianova). To inhibit the unoccupied binding sites of Cy2-anti-rabbit IgG, the sections were incubated for 3 h with 50% rabbit antiserum. The sections were subsequently subjected to an overnight incubation with 10 mg/mL rabbit anti-GFAP antibodies. The sections were subjected to a 1 h incubation period with Cy3-anti-digoxin (20 mg/mL; Dianova) to detect the presence of GFAP immune reactivity (Cobb et al., 2016; Saur et al., 2016; Marques et al., 2023).

Morphological attributes of hippocampal neurons were examined under a stereological system manufactured by ZEISS at 2.5× magnification. The experimental design incorporated a predetermined area sampling fraction of 2% to ensure representative data collection.

In order to accurately quantify the number of GFAP^+^ cells (astrocytes) within a given tissue sample, a “guard zone” with a width of 3 μm was implemented at the uppermost surface of the sections. Subsequently, the number of GFAP^+^ cells was counted within a depth of 15 μm below the guard zone, which corresponds to the height of the dissector. The estimation of the total number of astrocytes (*N*) was conducted using the formula: *n* = Σ*Q*^−^ × 1/ssf × 1/asf × 1/hsf. Σ*Q*^−^ is denoted as the quantitative representation of GFAP^+^ cells observed in the specimens. The section sampling fraction (ssf), area sampling fraction (asf), and height sampling fraction (hsf) are used to account for the respective fractions of the total sample that were analyzed in terms of sections, areas, and heights (Olude et al., 2015).

###### 2.5.4.3.1. Data analysis and statistical measures

All statistical analysis was performed in the Paleontological Statistics Software Package (PAST-4) and the significance level was set at *p* < 0.05. Results were expressed as mean ± SEM. To address the issue of multiple comparisons and to control the familywise error rate, the adjusted significance level was set at *p* < 0.0018. Comparisons within the groups on behavioral tests and astrocytes were done by one-way ANOVA. Power analysis was conducted to test for the effect size in the results. Furthermore, after the ANOVA result, if the results indicated a significant overall effect, we performed a *post hoc* Tukey’s test to further analyze and compare the means of different groups. Image-J was used to quantify hippocampal astrocyte density (HAD) and process intersections. The data on the effects on cognition, memory, and motor were recorded and plotted in bar graphs, box plots, and radar plots.

## 3. Results

### 3.1. ELS and schizophrenia alleviated motor on the SD using open field test

#### 3.1.1. Results on the distance covered

**Figure 4:**
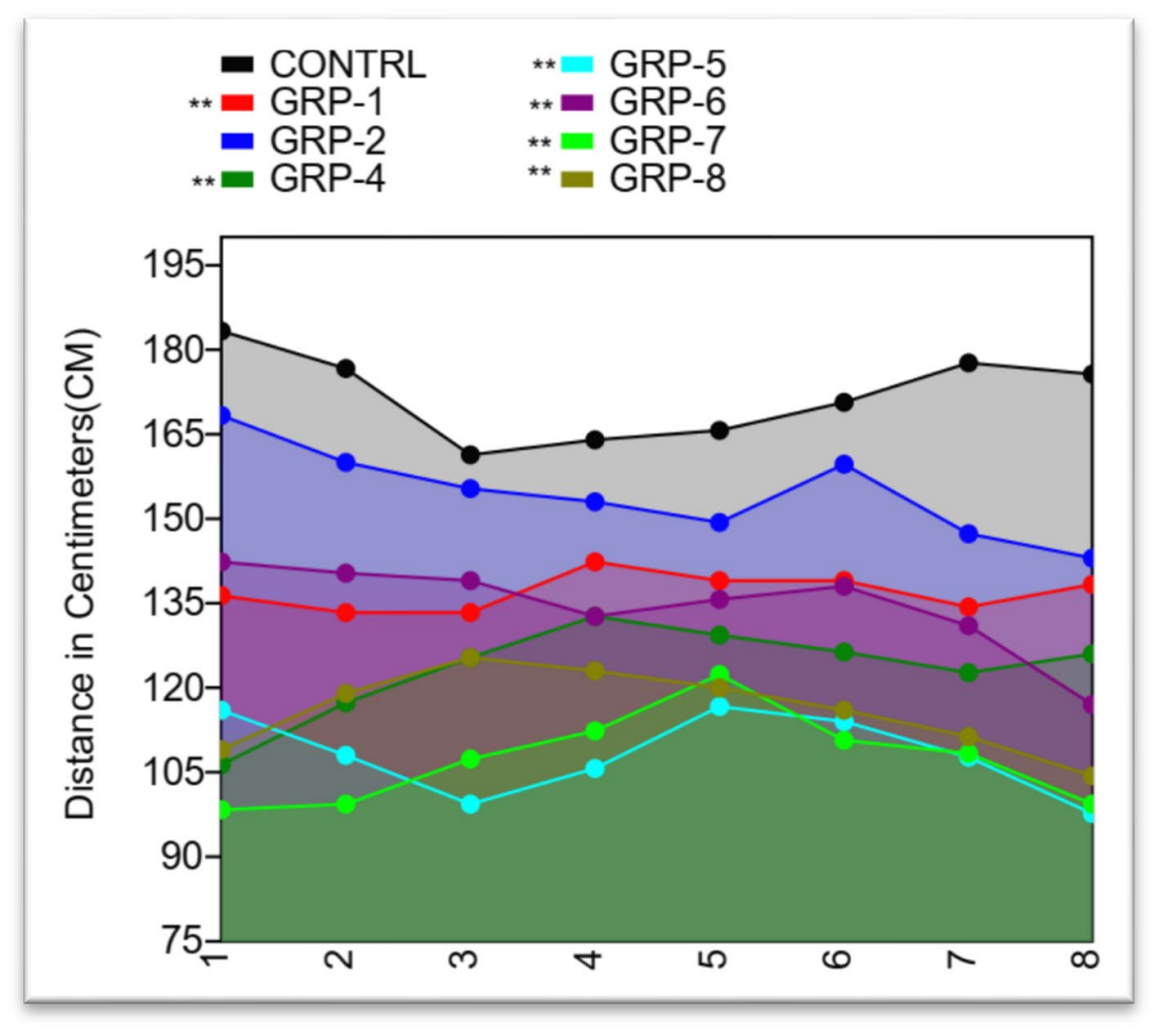
Distance covered by Sprague Dawley rats in an open field test. The line graph shows the mean distance covered by rats in each of the eight treatment groups plotted against the standard deviation (SD). The distance covered by rats ranged from a minimum of 88 to a maximum of 200 cm. The mean distance covered ranged from 107.25 to 171.875 cm, with a coefficient of variation ranging from 7.04% to 14.00%. Statistical significance was determined using one-way ANOVA, with Bonferroni correction (^*^p-value <0.05; ^**^p-value <0.01).

The OFT is an important tool for assessing anxiety-like behavior and locomotor activity in rodents. The analysis of variance conducted (control group 1, group 2, group 4, group 5, group 6, group 7, and group 8) revealed a significant difference in distance covered (*p* < 0.001), indicating a decrease in motor performance in the stressed groups [control vs. group 1 (prenatal stress and SZ pups), *p* < 0.001; control vs. group 4 (maternally separated pups-MSP), *p* < 0.001; control vs. group 5 (MSP and schizophrenic parents), *p* < 0.001; control vs. group 6 (SZ parents only), *p* < 0.001; control vs. group 7 (SZ parents and pups), *p* < 0.001; and control vs. group 8 (normal parents but SZ pups), *p* < 0.001]. There was also a significant difference between group 5 and group 6 (*p* = 0.1676) as well as between group 6 and group 7 (*p* = 0.1192) (see Figure 4).

#### 3.1.2. Results on number of entries into the center and peripheral zones

In order to conduct a comprehensive examination of the effects of ELS on anxiety-like behavior and general locomotor activity, we conducted additional measurements by recording the frequency of entries into both the central and peripheral zones (PZ) of the experimental field. The statistical analysis revealed a significant difference between groups [control (non-stressed) group vs. groups 1, 4, 5, and 6 (*p* < 0.001), group 2 and group 4 (*p* > 0.05), and group 2 vs. group 5 (*p* = 0.056)]. In contrast, the observed data did not reveal any statistically significant distinction between the groups subjected to stress and the group diagnosed with SZ (group 8) (*p* > 0.05).

On the duration of time spent in the center zone. The findings of our study demonstrated a significant influence of early life stress (ELS) and parental psychopathology on the time that rats spent in the center zone across the different groups. The statistical analysis revealed a significant effect (*p* < 0.001).

**Figure 5:**
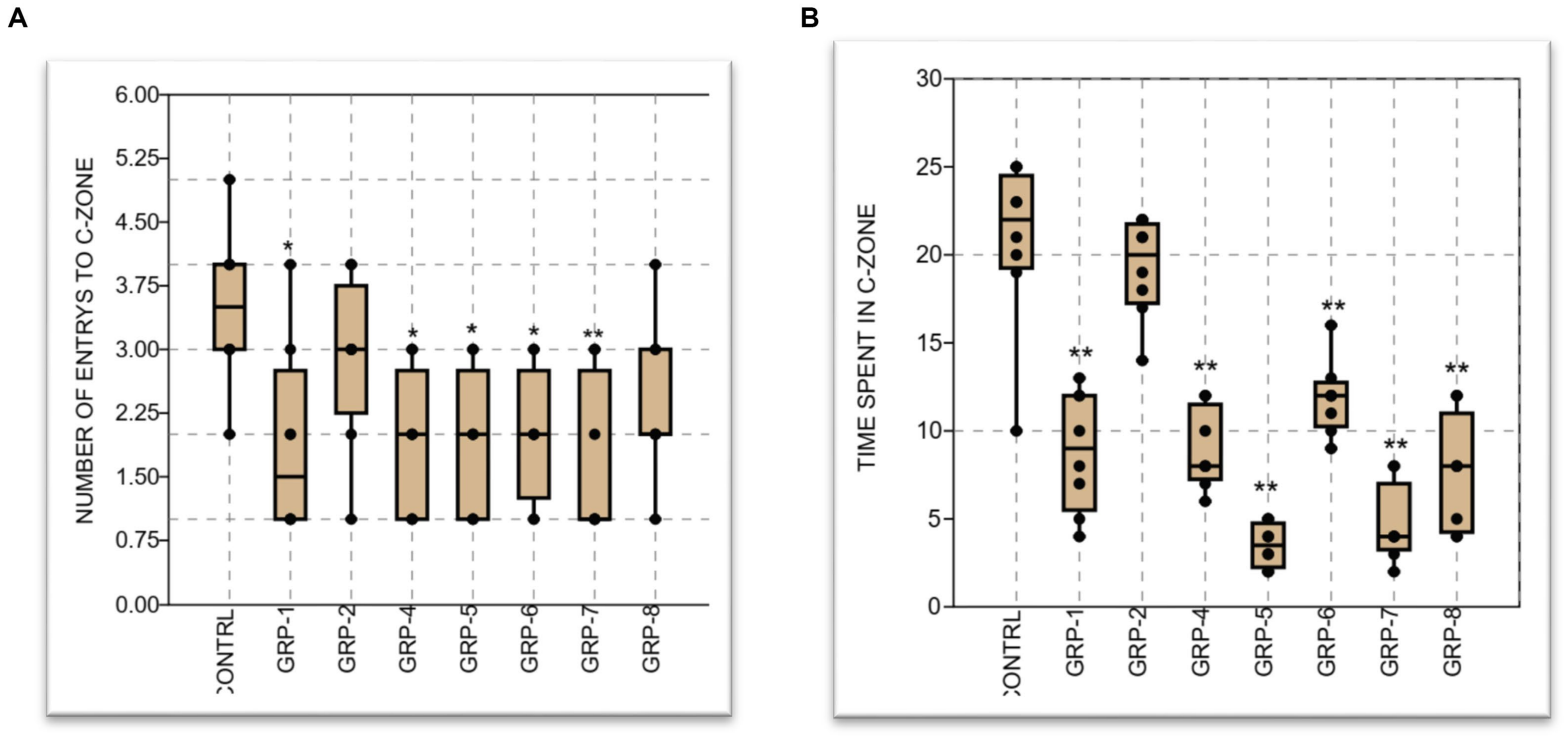
**(A)** The graph represents the mean number of entries to the center of the OPT by eight groups of animals: a control group and groups 1–8, respectively. The control group had a mean value of 3.5 entries, while groups 1–8 had mean values ranging from 1.875 to 2.875 entries. Graph **(B)** displays the mean time spent at the center, along with the standard error of the mean (SEM). The control group spent the most time at the center with a mean of 20.75 min, while the experimental groups had lower mean times ranging from 8.875 to 4.5 min. Statistical significance was determined using one-way ANOVA, with Bonferroni correction (^*^p-value <0.05; ^**^p-value <0.01).

There was a significant increase in anxiety-like behavior when comparing the control group to groups 1, 4, 5, 6, 7, and 8 (*p* < 0.001). However, the prenatally stressed group (group 2) did not exhibit a significant difference in anxiety-like behavior compared to the control group (*p* < 0.05). Furthermore, there was a significant difference between group 7 and group 8, (*p* < 0.001), group 6 vs. group 7 (*p* < 0.001), and group 5 vs. group 6 and group 8 (*p* < 0.001) (see Figure 5).

**Figure 6:**
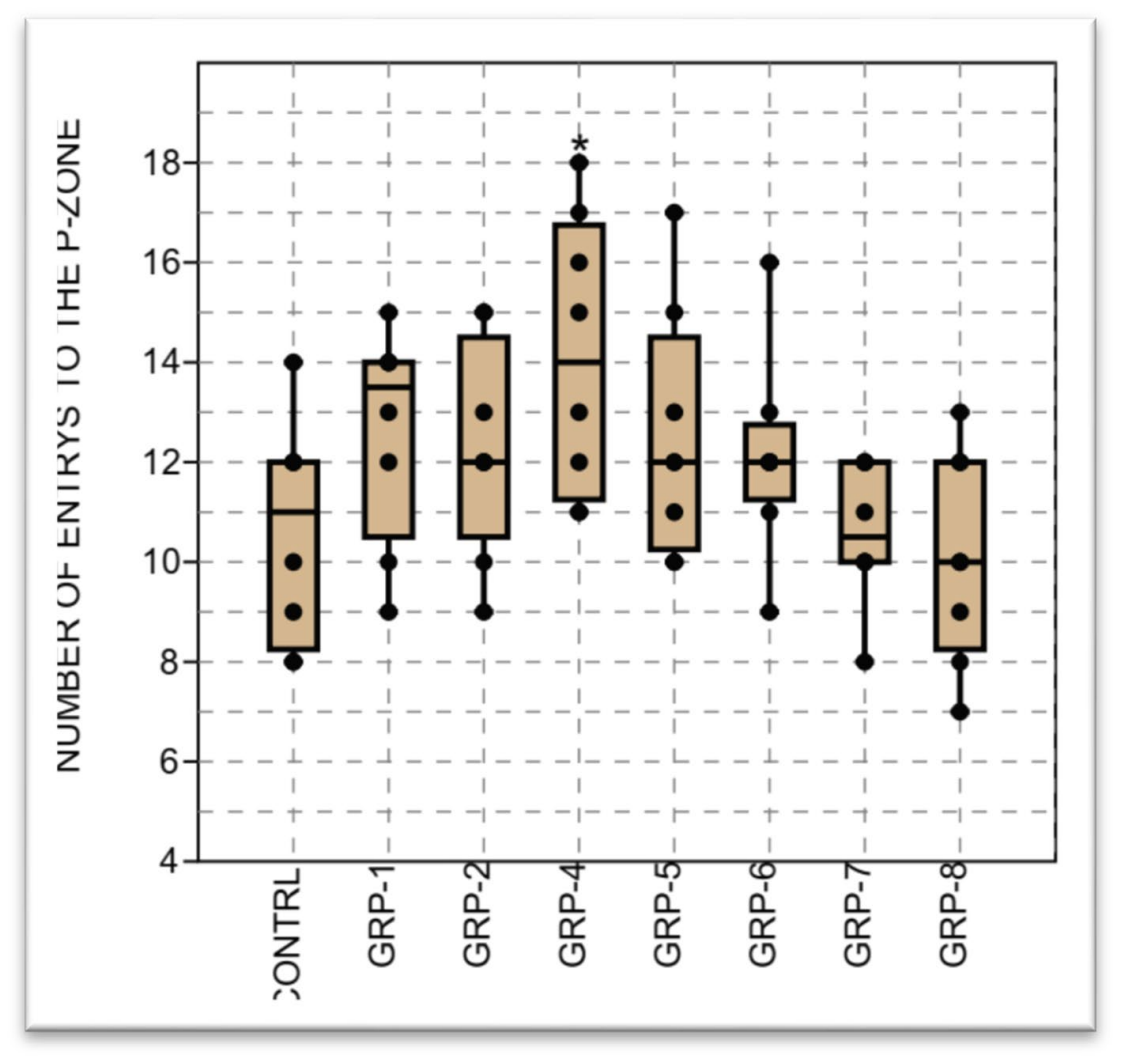
The figure represents the results of the open field test assessing the number of entries to the peripheral zone in different groups. The data shows a significant difference in the sample medians (*H* = 14.51, *p* = 0.035). Statistical significance was determined using one-way ANOVA, with Bonferroni correction (^*^p-value <0.05; ^**^p-value <0.01).

**Figure 7:**
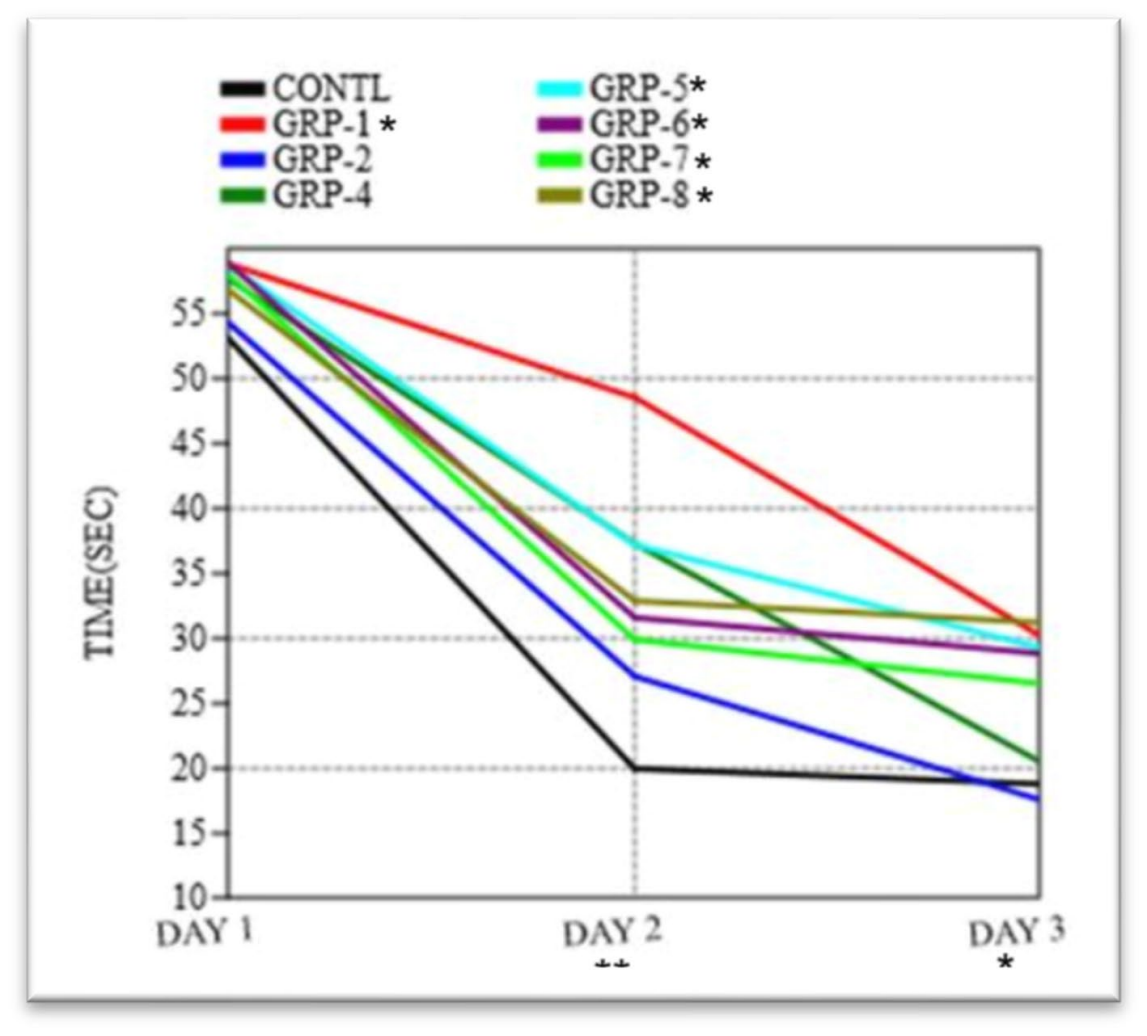
Time taken to find the platform in the Morris water maze test. The graph shows the mean time taken (in seconds) for rats to find the platform in the water maze. The ANOVA showed a significant difference between the groups [*F* (7, 16) = 0.2404, *p* = 0.9683]. Statistical significance was determined using one-way ANOVA, with Bonferroni correction (^*^p-value <0.05; ^**^p-value <0.01).

**Figure 8:**
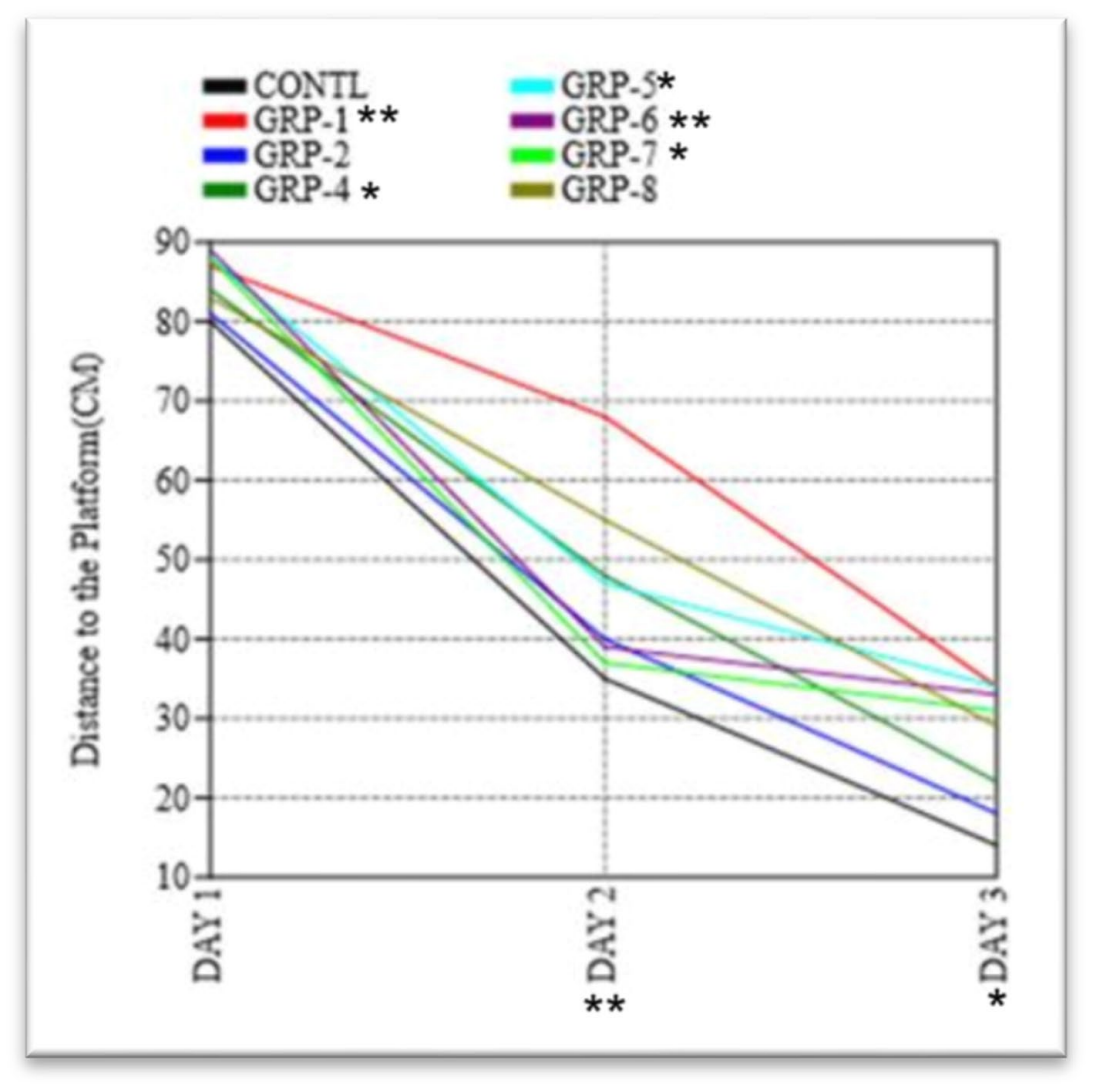
The figure shows the results of the Morris water maze experiment on the distance traveled by rats to find the hidden platform. Statistical significance was determined using one-way ANOVA, with Bonferroni correction (^*^p-value <0.05; ^**^p-value <0.01).

Finally, the analysis of variance on the number of entries into the PZ in this study showed a significant difference in the mean in the groups (*p* < 0.001). There was a significant difference in the number of entries to the peripheral zone (PZ) between the control (non-stressed) and the rest of the groups i.e., control vs. groups 1 (*p* < 0.001); control vs. group 2 (*p*  = 0.018); control vs. group 4; control vs. group 5; group 6; group 7; group 8 (*p* <0.001). In addition, group 2 was also significantly different from group 6 (*p*  = 0.017) and group 7 (*p*  = 0.033) (see Figures 6–8).

### 3.2. Early life stress impairs memory and cognition (MWM)

**Figure 9:**
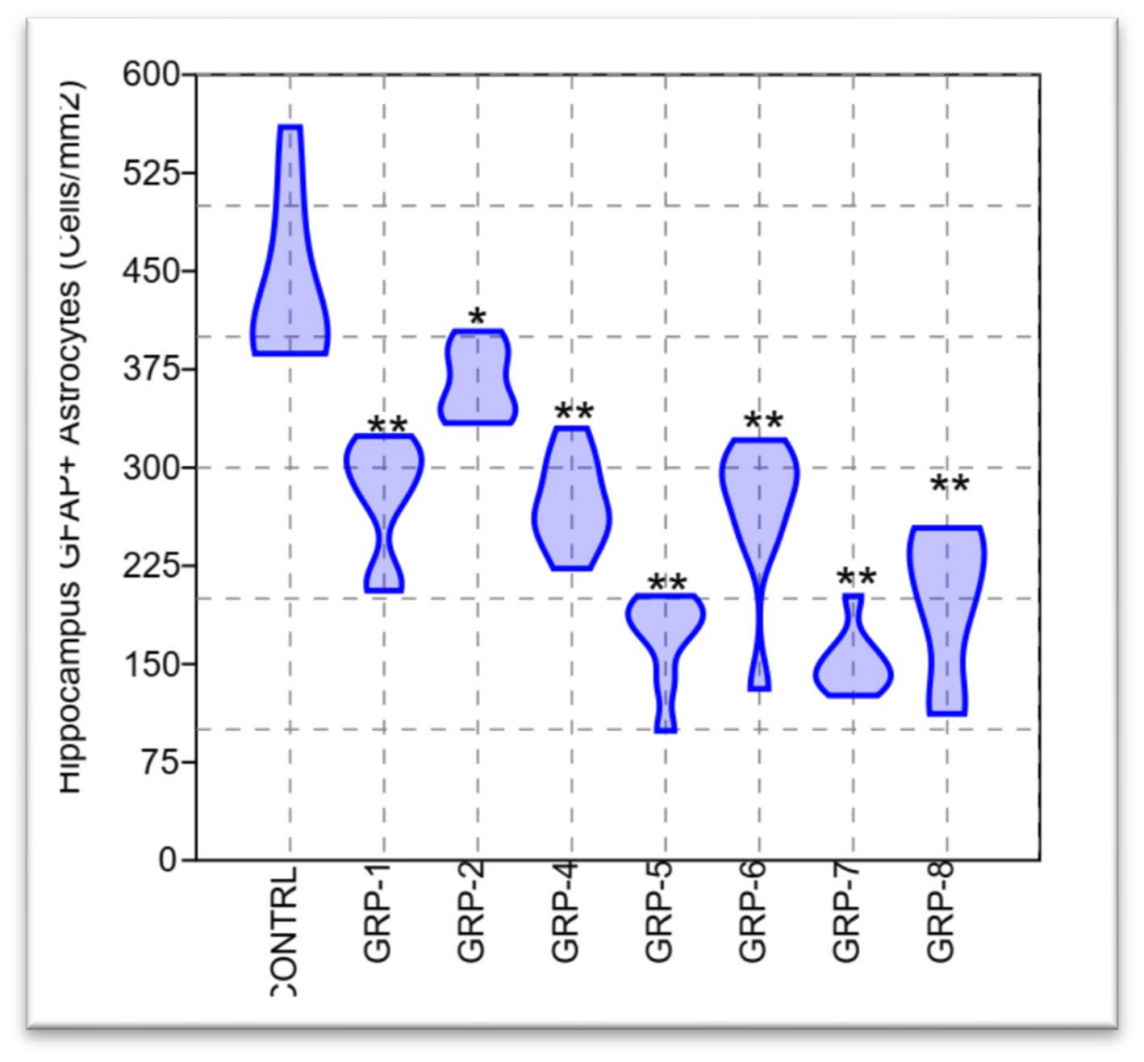
Violin plot of the astrocyte density in the hippocampus for eight treatment groups. Statistical analysis revealed significant differences between the groups in both mean and median density [*F* (7, 56) = 36.75, *p* < 0.001; Hc = 52.03, *p* < 0.001]. Statistical significance was determined using one-way ANOVA, with Bonferroni correction (^*^p-value <0.05; ^**^p-value <0.01).

The present study aimed to investigate the impact of ELS and SZ on spatial learning and memory in the MWM paradigm. During the acquisition phase, a series of repeated measures were conducted to gather data and analyze the effects of the time taken to find the platform. The ANOVA conducted in this study demonstrated a statistically significant effect of the trial [*F* (5, 60) = 8.41, *p* < 0.001], suggesting that there was a noticeable improvement in performance across the different trials. The rats exhibited a notable reduction in the time taken to locate the concealed platform and traversed a shorter distance during repeated trials. These findings provide evidence for the acquisition of learning and spatial memory, as depicted in Figure 9. In the subsequent probe trial, a one-sample t-test was conducted to examine the animals’ time spent in the target quadrant. The results indicated a statistically significant difference as the animals spent a significantly higher proportion of time in the target quadrant compared to chance levels [*t* (15) = 4.62, *p* < 0.001 *ω*^2^ = 0.2348]. Furthermore, the observed animals demonstrated a notably higher frequency of traversals over the designated platform area [*t* (15) = 3.24, *p* < 0.01], suggesting a spatial inclination toward the quadrant containing the target.

In order to evaluate the extent of memory retention, a memory recall trial was executed to compare the performance across eight distinct groups. Our findings indicate that there were no statistically significant variations in the means observed among the different groups on day 1 (*p* = 0.9683). Significant variations in the means were observed among the groups on day 2 (*F* = 15.65, df = 99.21, *p* < 0.001 *ω*^2^ = 0.6348). There were significant differences in the duration required to locate the platform on day 2 and day 3 among the various experimental groups [control group vs. group 1 (*p* < 0.001), control vs. group 2 (*p* = 0.036), control vs. group 4 (*p* < 0.001), control vs. group 5 (*p* = 0.1972), control vs. group 6 (*p* < 0.001), and control vs. group 7 (*p* < 0.001)]. However, there was no difference between group 8 and the control group (*p* > 0.05).

In the course of the probe trial, there were no statistically significant disparities observed in the duration of time spent in the target quadrant across the various groups [*F* (2, 24) ¼ 2.332, *p* > 0.05; Figure 9]. Furthermore, there were no statistically significant variations in swimming speed observed among the eight experimental groups [*F* (2, 24) ¼ 3.077, *p* > 0.05] during the initial day of the training period. The analysis of the data indicated that there was no statistically significant variation in the average distances traveled by the different groups on the first day [*F* (7, 16) = 0.1249, *p* = 0.995 *ω*^2^ = 0.1348]. In the course of the experiment, it was observed that there existed a substantial disparity in the distance to the platform (DT) between the control group and various experimental groups on both days 2 and 3 [group 1 (*p* < 0.001), group 2 (*p* < 0.001), group 4 (*p* < 0.001), group 5, group 6, group 7, and group 8 (*p* < 0.001)]. These findings indicate a significant divergence in the DT measurements between the control group and the aforementioned experimental groups. The results of the experiment indicate that there was no statistically significant difference in the time taken to find the platform among groups 4, 5, and 6 (*p* > 0.05). However, group 8 exhibited a significant difference in the time taken to find the platform compared to group 7 (*p* = 0.007).

### 3.3. Early life stress-induced hippocampal astrocytes volume loss

We used the sampling scheme in Table 2 to quantify astrocyte density in the hippocampus. The table includes the number of animals in each group, the sampling method employed (random), the number of sections sampled, the section thickness in micrometers, the number of counting frames sampled, and the number of GFAP^+^ cells sampled.

**Table 2:** Sampling scheme and GFAP^+^ cell analysis in the hippocampus.

Group	Number of sections sampled	Section thickness (μm)	Number of counting frames sampled	Total number of GFAP^+^ cells	Sampling method
Control	8	24.41 ± 1.55	4	3,506 ± 64	Random
Group 1	8	24.41 ± 1.55	5	2,231 ± 44	Random
Group 2	8	24.41 ± 1.55	4	2,920 ± 27	Random
Group 3	8	24.41 ± 1.55	5	2,186 ± 34	Random
Group 4	8	24.41 ± 1.55	4	1,359 ± 35	Random
Group 5	8	24.41 ± 1.55	5	2,126 ± 60	Random
Group 6	4	24.41 ± 1.55	4	1,210 ± 23	Random
Group 7	8	24.41 ± 1.55	4	1,593 ± 55	Random

Section thickness is represented as mean ± standard deviation (SD), whereas the number of counting frames and the numbers of sampled GFAP^+^ cells are represented as mean with range in parentheses.

The present study aimed to investigate the potential variations in the density of GFAP^+^ astrocytes in the hippocampus across different experimental groups. To address the issue of multiple comparisons with eight groups, we applied the Bonferroni correction method, dividing the desired significance level (alpha) by the number of comparisons performed. The adjusted alpha level for each individual *post hoc* test was set at 0.05/28 (8 groups choose 2), resulting in an adjusted significance level of approximately *p* < 0.0018. Our findings revealed a statistically significant disparity between the groups [*F* (7, 56) = 36.75, *p* < 0.001 *ω*^2^ = 0.7963], indicating a substantial effect size. This finding suggests that a significant proportion, specifically 79.63%, of the observed variations in HAD can be ascribed to the distinctions among the groups subjected to ELS and those with parental SZ. The *post hoc* analysis revealed statistically significant differences (*p* < 0.05) among the means of all groups, with the exception of the comparisons between groups 1 and 4 (*p* = 1), groups 1 and 6 (*p* = 0.999), and groups 5 and 7 (*p* = 0.002). There was a significant difference between group 7 and group 8 (*p* = 0.043).

#### 3.3.1. Hippocampal astrocytes processes intersections (lateral and central)

The analysis conducted on the lateral processes revealed significant findings regarding the test for equal means. Our results indicate that the control group exhibited statistically significant differences when compared to the other groups [group 1 (*p* < 0.001), group 2 (*p* = 0.031), group 4 (*p* < 0.001), group 5 (*p* < 0.001), group 6 (*p* < 0.001), group 7 (*p* < 0.001), and group 8 (*p* < 0.001)]. There was no significant difference between group 7 and group 8 (*p* = 0.004).

**Figure 10:**
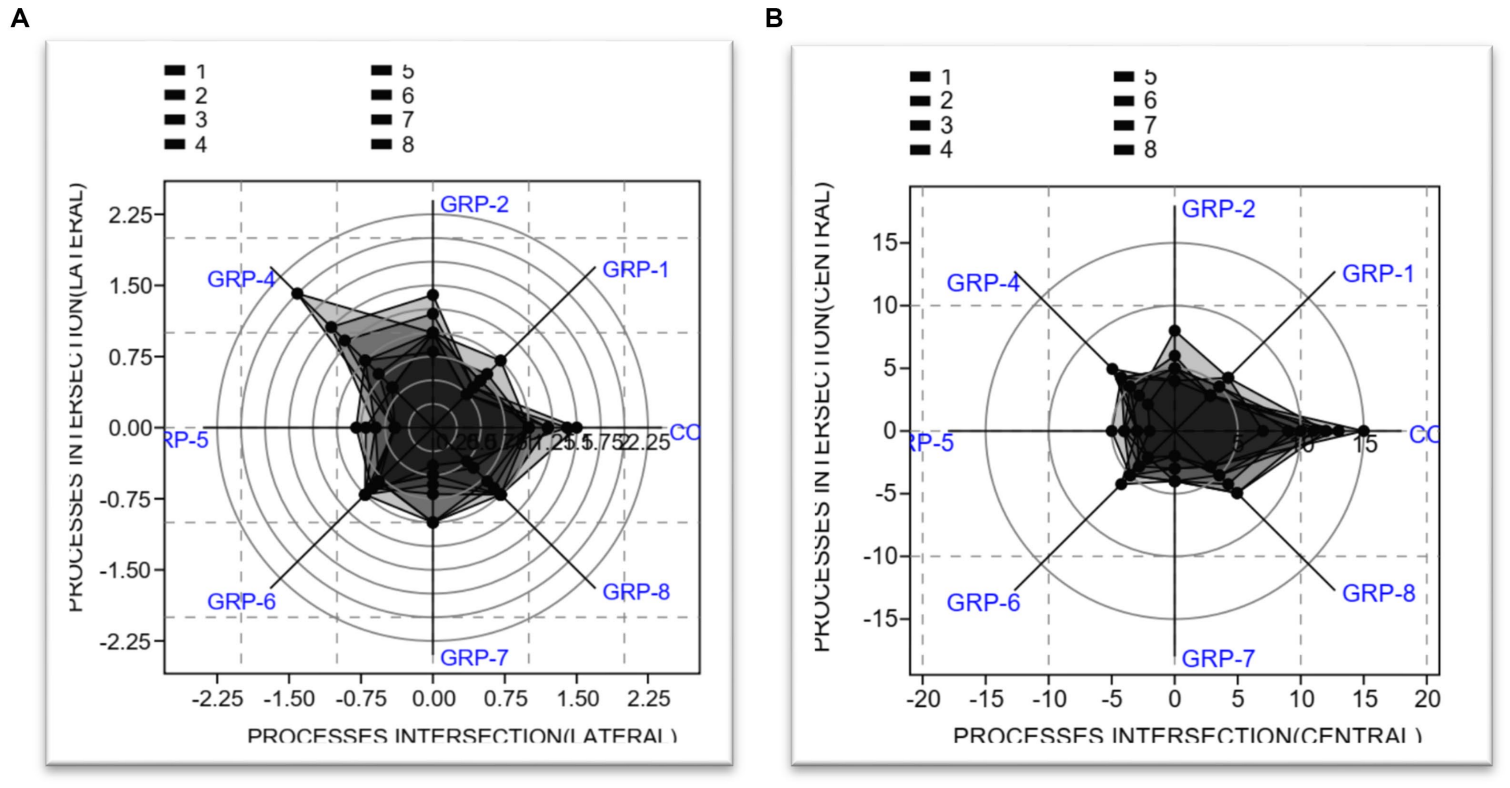
Analysis of astrocyte processes intersection. **(A)** Radar chart showing the lateral process. **(B)** Radar chart showing the central process. The two radar charts show the mean length of lateral processes, central processes, and total processes in primary astrocytes. The total length of astrocytic processes was the sum of lateral and central processes and had a mean length of 0.6 μm.

The present study also investigated the central processes intersection and its implications. The obtained results from the ANOVA indicated a statistically significant effect [*F* (7, 56) = 4.675, *p* < 0.001 *ω*^2^ = 0.3804]. The results of the *post hoc* test comparisons indicate that there are significant differences in means between the control group (non-stressed) and groups 1, 2, 4, 5, 6, 7, and 8, *p* < 0.001 (see Figure 10).

**Figure 11:**
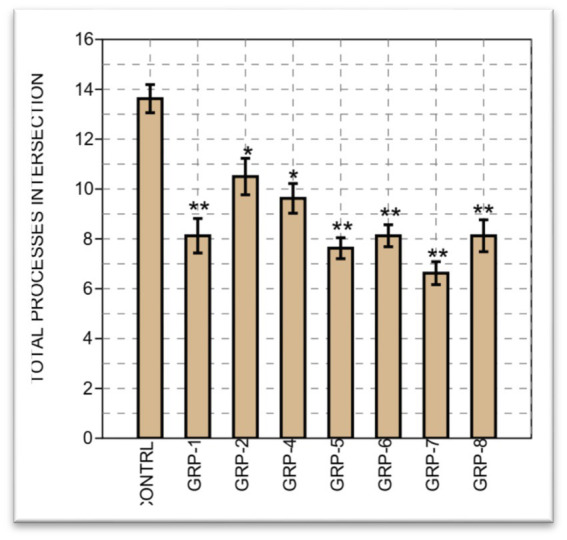
The bar graph illustrates the total astrocyte intersection processes across eight groups, accompanied by standard errors. Each bar represents the mean value of astrocyte intersection processes, while the error bars indicate the standard errors. The group labels are displayed on the *x*-axis, and the *y*-axis represents the total number of astrocyte intersection processes. Statistical significance was determined using one-way ANOVA, with Bonferroni correction (^*^p-value <0.05; ^**^p-value <0.01).

Finally, on the total processes intersection, we compared the means of eight different groups. Between groups, a significant difference was observed, with [*F* (7, 56) =8.714, *p* < 0.001 *ω*^2^ = 0.4576]. A significant difference was observed between most groups, except between group 1 and groups 5 (*p* = 0.998), 6 (*p* = 1), 7 (*p* = 0.625), and 8 (*p* = 1). Finally, we performed a two-sample t-test to compare the means between group 7 and group 8. The results of the t-test revealed a *t*-value of 1.9042, with a corresponding (*p* = 0.077). Considering that we obtained (*p* = 0.077), which is close to the significance threshold, there is marginal evidence to suggest a significant difference between the means of the groups on the total processes intersections (see Figure 11).

## 4. Discussion

In the present study, we explored the impact of early life stress (MS and GS) and parental SZ or their interactive nature on locomotor activity, anxiety-like behavior, exploratory tendencies, and spatial memory. Our study aimed to unravel the complex relationships between these stressors and cognitive outcomes and also the potential underlying mechanisms. The experimental groups included the control group 1 (non-stressed), group 2 (prenatal stress and SZ pups), group 3 (prenatal stress), group 4 (MS), group 5 (MS and SZ parents), group 6 (SZ parents), group 7 (SZ parents and pups), and group 8 (SZ pups only). Varied ELS, including MS and GS, exerted distinct effects on SD, resulting in a marked elevation in anxiety-like behavior and a concurrent decrease in locomotor activity. Moreover, spatial memory capabilities exhibited a notable reduction in response to these stressors. In the context of maternal SZ, heightened anxiety-like behavior and evident psychomotor retardation were observed, along with a concomitant decline in spatial memory function. Interestingly, the influence of standard MC appeared to have a reversal effect on anxiety-like responses, coupled with an enhancement of motor activity, and spatial memory performance became apparent.

The OFT serves as a valuable tool to assess anxiety-like behavior and locomotor (Seibenhener and Wooten, 2015). In this present study, significant differences were observed in the locomotion using OFT indicating reduced motor performance across the groups. These findings suggest that both ELS and parental psychopathology independently contribute to motor deficits in the rat model (*p* < 0.05), with group 2-prenatal stress (GS) exhibiting the least pronounced impact on psychomotor functioning. Also, on the OFT, the researchers investigated the differences in time spent at the center zone among various groups, which is an indicator of the animal’s willingness to explore the environment and its anxiety behavior. A statistically significant difference (*p* < 0.05) was observed between the control group and the remaining groups (ELS and SZ). Specifically, SZ pups raised by SZ dams (group 7) exhibited increased anxiety and reduced exploratory behavior compared to the SZ pups that had normal parents (group 8) (*p* = 0.046). This suggests that good parenting (normal parents) may have a reversal effect on anxiety-like behavior and psychomotor retardation in the offspring affected by SZ. The findings imply that the environment and care provided by normal parents may play a crucial role in mitigating anxiety-related responses and improving motor behavior in the context of ELS and parental psychopathology. Furthermore, the researchers observed that groups subjected to differential MS and SZ (groups 5, 6, and 7) displayed a significantly reduced inclination toward exploratory behavior. The present findings suggest that ELS and parental psychopathology, specifically SZ, exert a lasting influence on the emergence of anxiety and depressive behaviors later in life.

Previous research has highlighted the impact of ELS such as MS and CUS on motor activity and anxiety-like behavior in rodents (Rana et al., 2015; Alves et al., 2022). For instance, the study by Rana et al. (2015) found that rats exposed to early-life MS showed an increase in anxiety- and depressive-like behaviors, leading to decreased exploration in the OFT, which is consistent with our present findings. In addition, numerous studies have also demonstrated that MS and CUS can lead to alterations in the HPA axis and the limbic system, resulting in heightened anxiety-like behaviors and changes in locomotor activity (van Bodegom et al., 2017; Walker et al., 2018; Orso et al., 2020). To be specific, van Bodegom et al. (2017) reported that ELS induced by prenatal stressors, MS, or the limited nesting model can have significant consequences on the function of the HPA axis in adulthood. The effects of ELS on the HPA-axis function are dependent on various factors, including the developmental time window of exposure, the sex of the offspring, and the developmental stage at which effects are assessed. Furthermore, ELS results in HPA-axis hyper-reactivity in adulthood are similar to what is observed in studies on severe depression in humans. This provides empirical evidence on the pathophysiology of anxiety and psychomotor retardation as observed in our study. Our results are in line with these studies; hence, they reinforce the validity of the rat model in investigating the behavioral consequences of ELS.

As far as we understand, there have been no published studies modeling the impact of parental SZ in rodents. However, over the past few years, a growing body of evidence has lent support to the notion that the malfunctioning of the glutamatergic system constitutes a fundamental pathological alteration observed in SZ. Consequently, the inhibition of the NMDA receptor through the use of non-competitive antagonists like ketamine or PCP triggers the emergence of delusions and hallucinations in individuals who are otherwise in a healthy state, mirroring the symptomatic profile frequently associated with SZ model in rodents (Krystal et al., 1994; Kapur and Seeman, 2002). Nevertheless, comparisons drawn from unidirectional studies in humans have revealed effects on offspring later in life linked to parental SZ. Children from SZ parents have a higher likelihood of developing specific psychiatric disorders such as anxiety, depression, and cognitive impairments during their lifetime (van der Pol et al., 2016; Weijers et al., 2018; Steenhoff et al., 2021). Findings from Vandeleur et al. (2012) indicated that offspring of individuals with bipolar disorder (BPD) and major depressive disorder (MDD) have elevated rates of mood and anxiety disorders compared to control groups. This further supported (Rasic et al., 2014) meta-analysis that reported that the offspring of parents with severe mental illness (SMI) are at increased risk for a range of psychiatric disorders, and one-third of them may develop an SMI by early adulthood.

The MWM test was employed to assess spatial memory and cognitive functioning in the different experimental groups. During the acquisition phase, the researchers observed significant differences in the performance of the experimental groups compared to the control group 1 (non-stressed). The stressed groups showed prolonged latencies and increased path lengths compared to the control group, indicating impaired spatial learning. GS and SZ pups exhibited the most substantial impairment in spatial learning (*p* < 0.001) compared to the control group. This finding suggests that the combination of prenatal stress and SZ leads to more severe cognitive deficits in the offspring. Similarly, groups that were subjected to prenatal stress, MS, MS with SZ parents, and SZ pups only, also displayed significant impairments in spatial learning compared to the control group (*p* < 0.001). These results indicate that ELS, irrespective of the presence of parental psychopathology, is sufficient to impair spatial learning.

To assess spatial memory retention, a probe trial was conducted on the third day, during which the platform was removed from the pool. The control group showed a significant preference for the target quadrant, spending more time (*p* < 0.001) and making more crossings (*p* < 0.001) in this area compared to other quadrants. In contrast, all stressed groups displayed reduced preference for the target quadrant, spending significantly less time (*p* < 0.001) and making fewer crossings (*p* < 0.001) in this region. Parental SZ with SZ pups (group 7) exhibited the most severe deficits in spatial memory retention, displaying minimal preference for the target quadrant compared to the other stressed groups. These results indicate that differential ELS and parental psychopathology impair spatial memory retention in the rat model. To assess cognitive flexibility and adaptability, a reversal-learning task was conducted. The platform was moved to the opposite quadrant, and the rats were trained to locate the new platform location over three consecutive days. The stressed groups (group 2, group 3, group 4, group 5, and group 8) showed significant deficits in reversal learning compared to the control group (*p* < 0.001). Group 7 again exhibited the most profound cognitive inflexibility, as indicated by significantly longer latencies (*p* < 0.001) and path lengths (*p* < 0.001) compared to the control group. These results suggest that ELS and parental psychopathology not only impair initial spatial learning but also hinder the rats’ ability to adapt and relearn a new platform location during reversal learning.

In the realm of rodent behavior and cognitive tasks, mnemonic functions play a crucial role, encompassing both long-term and short-term working memory components. Working memory is believed to depend on sustained neural activity within an active network, while long-term memory and synaptic plasticity are instrumental in shaping the synaptic structure, thereby influencing the spectrum of potential states in memory processing (Sheynikhovich et al., 2023). The deficits observed during the reversal learning task suggest that ELS and parental psychopathology may impair cognitive flexibility and adaptability. The reduced ability to learn a new platform location indicates potential alterations in the rats’ executive functions and prefrontal cortex functioning, which are crucial for cognitive flexibility (Piao et al., 2017). Overall, the findings from the MWM provide compelling evidence of the adverse effects of ELS and parental psychopathology or PTSD on spatial learning, memory, and cognitive flexibility in the rat model. These results align with previous research, indicating that ELS can lead to hippocampal dysfunction and alterations in synaptic plasticity, ultimately contributing to cognitive deficits (Floresco et al., 2009; Popescu et al., 2023). As with any scientific study, our research has some limitations that merit consideration. The use of the rat model, while advantageous for controlled experiments, does not fully represent the complexity of human experiences and psychopathology. Additionally, the choice of specific behavioral tests may not fully capture all aspects of cognitive functioning.

The neurochemical analysis aimed to quantify the density of GFAP^+^ astrocytes in the hippocampus across the different experimental groups. Astrocytes are a crucial glial cell type involved in maintaining neural homeostasis and supporting cognitive functions. The findings from the neurochemical analysis revealed a statistically significant disparity in HAD among the different experimental groups. This substantial effect size can be attributed to the distinctions among the groups subjected to different ELS and those with parental SZ. These findings indicate that ELS and parental psychopathology independently influence hippocampal astrocyte density. The hippocampus is a brain region crucial for memory formation and spatial learning (Kemp et al., 2022). It is involved in the consolidation and retrieval of spatial memories, which are processes that were found to be impaired in the behavioral results. Astrocytes play a vital role in synaptic plasticity, neurotransmitter regulation, and maintenance of the brain’s microenvironment (Marathe et al., 2018; Kim et al., 2019). The observed reductions in hippocampal astrocyte density in the stressed groups may have implications for hippocampal function. Previous research has shown that chronic stress can lead to astrocyte atrophy and decreased astrocytic processes in the hippocampus, potentially compromising synaptic plasticity and cognitive functioning (Kaplan et al., 2018; Li et al., 2022).

The hippocampus is vulnerable to the effects of stress and plays a critical role in emotion regulation and memory processing (Kim et al., 2015; Hueston et al., 2017). Dysregulation of astrocyte function in the hippocampus may contribute to the cognitive deficits observed in these groups.

In addition to overall astrocyte density, the analysis focused on astrocytic processes, specifically lateral and central processes. Astrocytic processes are essential for enabling synaptic communication and supporting neuronal function (Chiareli et al., 2021). The results showed significant differences in the length of lateral and central processes between the control group and all other experimental groups. The observed alterations in astrocyte density and processes in the hippocampus have potential implications for cognitive function. Astrocytes are known to modulate synaptic transmission, regulate extracellular neurotransmitter levels, and influence synaptic plasticity (Henneberger and Petzold, 2015; Durkee and Araque, 2019). Changes in astrocyte function may affect synaptic communication and compromise the neural circuits responsible for cognitive processes. The hippocampus plays a critical role in spatial learning and memory (Apple et al., 2017; Toda et al., 2019). Reduced astrocyte density and altered astrocytic processes in the hippocampus may contribute to the deficits observed in the MWM (Jahanshahi et al., 2008; Sampedro-Piquero et al., 2014). Impaired astrocyte function could lead to disruptions in the hippocampal microenvironment and synaptic plasticity, impacting memory formation and spatial learning.

In summary, our research findings indicate a significant correlation between ELS and SZ, with notable effects on motor function, memory, learning ability, and the volume of GFAP astrocytes. Henceforth, the findings of our study have demonstrated the successful establishment of our depression animal model. Furthermore, our investigation has revealed a significant association between Neuro-Psycho-biological dysregulation and the observed outcomes.

## 5. Conclusion

In conclusion, our study utilizing a rat model provides significant insights into the impact of early life stress and parental psychopathology on motor and memory cognitive functioning. The results demonstrate that both stressors independently contribute to motor and cognitive deficits in the rat model. The findings highlight the complex and multifaceted nature of the relationships between early life experiences, parental psychopathology, and cognitive development. Importantly, our study introduces a novel perspective by demonstrating that positive parenting interventions can effectively enhance cognitive function and stimulate the hippocampus, contributing to regenerative astrocyte gliosis. Moving forward, further research is warranted to elucidate the underlying molecular mechanisms and to develop effective therapeutic strategies to mitigate the adverse effects of these stressors on cognitive outcomes. Taken together, this novel differential early life manipulation will be able to contribute to a clearer understanding of the negative effects of ELS and parental SZ symptoms on brain health.

## Data availability statement

The original contributions presented in the study are included in the article/supplementary material, further inquiries can be directed to the corresponding author.

## Ethics statement

The animal study was approved by Institutional Animal Use and Care Committee of the School of Laboratory Medicine and Medical Science at UKZN. The study protocol was assigned the reference number AREC/00003119/2021.

## Author contributions

FO was responsible for managing all literature searches. FO and TM collaborated on the initial draft of the paper and providing substantial input in the form of text passages and revisions for significant intellectual content. All authors contributed to the article and approved the submitted version.

## Abbreviations

ACE: Adverse Childhood Experiences
MDD: Major Depressive Disorder or Depression
ELS: Early Life Stress
SZ: Schizophrenia
MS: Maternal Separation
DL: Developmental Impact
CNS: Central Nervous System
HPA: Hypothalamic-Pituitary-Adrenal
PFC: Prefrontal Cortex
PTSD: Post-Traumatic Stress Disorder
